# Effect of Resistant Dextrin on the Functional, Thermal and Structural Properties of Cooked Chinese Rice

**DOI:** 10.3390/gels12060516

**Published:** 2026-06-10

**Authors:** Ruijun Chen, Qiuling Tang, Shiyu Chang, Barbara Conti, Xingjun Li

**Affiliations:** 1 Academy of National Food and Strategic Reserves Administration, National Engineering Research Center for Grain Storage and Transportation, Beijing 102209, China; ruijunchen1@126.com (R.C.); csy@ags.ac.cn (S.C.); 2 College of Food Science and Engineering, Wuhan Polytechnic University, Wuhan 430048, China; tangqiuling1145@163.com; 3 Department of Agriculture, Food and Environment, University of Pisa, Via del Borghetto 80, 56124 Pisa, Italy; barbara.conti@unipi.it

**Keywords:** cooked rice, gel-like structure, univariate general linear model, principal component analysis, amylose recrystallisation, gelatinisation

## Abstract

This study added two types of resistant dextrin (RD), i.e., Bailong (BL) and Luo Gaite (LGT)) to a Japonica (cv. RXY) and an early indica (cv. IP44) rice during cooking and analysed the functional and structural properties of the cooked rice. Compared with no RD addition, 3–10% RD addition induced a declinein cooking time and an incrementin gruel solid loss. Further, 3–10% RD addition increased the hardness, chewiness, and springiness of cooked rice but decreased the cohesiveness. With increases in the added RD amount, the smell, structural appearance, palatability, taste, cool rice texture, and total score of the cooked rice all increased; the peak time and pasting temperature increased, but the peak, final, breakdown, and setback viscosities all significantly decreased. The enthalpy, conclusion temperature of gelatinisation, and gelatinisation peak width and height all decreased with increasing RD amount, but the peak temperature of gelatinisation increased. The addition of 3–7% RD did not change amylopectin ageing, but 10% RD significantly increased amylopectin ageing. RD addition reduced the protein weakness degree and starch breakdown torque of rice doughbut appeared to increase the amorphous and crystalline regions of cooked rice. The addition of 10% BL or LGT induced the formation of α-helix and random coil secondary protein structures in cooked rice, with optimal cooking properties and total sensory score. Microstructure analysis further showed that low-viscous RD induced the formation of new gel-like structures. In conclusion, 3–10% RD addition in cooking rice decreases amylose recrystallisation, weakens the protein structure, and induces new gel-like structures, enhancing the hardness, chewiness, adhesiveness, springiness, and sensory score of cooked rice. This study is useful for developing functionalcooked rice.

## 1. Introduction

Resistant dextrin (RD), processed from starch via enzymatic hydrolysis or acid–heat treatment, possesses a branched structure with varying glycosidic linkages, such as α-1,2; α-1,3;β-1,2; β-1,3; β-1,4; and β-1,6 glycosidic linkages [1,2,3]. It offers nutritional benefits, including an approximate 70% fibre content, a low glycaemic index, and a low caloric value (0.5 kcal/g) [4]. RD is generally recognised as safe (GRAS) by the American Food and Drug Administration (FDA), with a safe daily intake of up to 45 g [5,6]. The unique linkages, including the α-1,2 and β-1,6 glycosidic linkages, in RD make it resistant to digestion in the upper gastrointestinal tract, allowing it to modulate postprandial satiety and the glycaemic response [7]. Thus, it can be utilised in healthy snacks, cakes, dairy products, various beverages, confectionery products, and meat products [8,9]. The Ministry of Health of China announced in 2012 that resistant dextrin is a general food, and a Light Industry Standard was issued on 29 March 2024 [10]. China’s resistant dextrin production was 60,000 tons in 2021, and it is predicted to reach 80,000 tons in 2026, with a wide range of applications, including in health care products (accounting for 38.7% of all applications) and functional foods (18%) [11]. However, research on the addition of RD during rice cooking is lacking.

When dextrin-type fibres are added to foods, the rheological behaviour and dough spreadability during baking, as well as the penetration resistance, are like those when sucrose is added, as RD exhibits a plasticising effect similar to that induced by sucrose, as compared to inulin-type dietary fibres [12]. Yu et al. [7] added RD to dough and found that low glycaemic index (GI) cookies exhibited a higher dietary fibre content without affecting the palatability of the cookies. Current reports mainly focus on inulin’s impact on starch crystallinity, gelation behaviour, and digestion performance, with little emphasis on the comparative effects of RD.

Cooked white rice is a high-GI cereal food, with a GI range of 73–92.4 [13]. Many researchers have demonstrated that long-term consumption of cooked white rice is related to a relatively high incidence of type II diabetes in some Asian countries [14]. At present, the addition of natural or synthetic dietary fibre to staple food is recommended as the primary approach to supplementing the daily dietary fibre intake of sub-healthy individuals. We hypothesise that the incorporation of RD during the cooking of rice might offer an approach to achieve functional cooked rice because RD is a starch-based, low-calorie, fibre-rich ingredient with verified prebiotic properties. In addition, we also hypothesise that the low-viscosity characteristic of resistant dextrin (approximately 0.01 Pa·s for a 30% aqueous solution [3]) might help to maintain stability after starch gelatinisation and possibly reduce retrogradation. While RD has been explored in beverages, gummies, extruded candy, and baked goods [15,16], its application during rice cooking has not been studied. Thus, this study uniquely evaluated RD’s functionality within the specific matrix, concentrating on its impact on the texture, gelation, and overall product quality of rice when added during cooking. A Univariate General Linear Model (UGLM) in SPSS (Statistical Product and Service Solutions) software is a statistical framework for analysing the linear relationship between a single continuous dependent variable and multiple predictors (including both categorical and continuous variables). It is widely used in analysis of variance, analysis of covariance, and multiple regression [17]. UGLM was employed to thoroughly assess the effects of different concentrations of RD on the physicochemical, texture, and sensory properties of cooked rice. The primary aim of this study was to explore the potential for achieving dietary-fibre-rich functional cooked rice through the addition of RD and to determine the optimum formulation based on the key quality parameters. This study will provide scientific insight and practical strategies for promoting fibre-enriched cooked rice innovations.

## 2. Results and Discussion

### 2.1. Effect of RD Levels on the Cooking Process of Two Varieties of Rice

Milled japonica rice (RXY) and an early indica rice (IP44) were used in the experiments; BL and LGT powders at weight ratios of 0%, 3%, 5%, 7%, and 10% were added to the two varieties of rice grains to obtain the test samples. Compared with IP44 rice, RXY rice had a shorter cooking time, lower water absorption rate, and greater gruel solid loss (Table 1). Compared with no added RD, both LB and LGT addition significantly lessened the cooking time of the two varieties of rice but increased the gruel solid loss. Compared with no RD addition, the cooking time of rice significantly decreased with increasing amounts of added RD, and the gruel solid loss increased significantly. The addition of 3% and 10% PD significantly reduced the rate of water absorption in rice during cooking. The decrease in cooking time and increase in gruel solid loss induced by 3–10% RD addition during rice cooking are similar to the results obtained with polydextrose addition [18].

### 2.2. Effect of RD Levels on the Textural Properties of Two Varieties of Cooked Rice

Compared with cooked IP44 rice, cooked RXY rice had lower hardness, resilience, cohesiveness, gumminess, and chewiness but higher adhesive force and adhesiveness (Table 2). Compared to no RD addition, BL and LGT addition increased the hardness, adhesiveness, adhesive force, gumminess, chewiness, and springiness parameters of cooked rice but decreased the resilience and cohesiveness values. Compared with BL addition, LGT addition significantly reduced the hardness, adhesiveness, adhesive force, gumminess, cohesiveness, and chewiness parameters of cooked white rice. With increases in the amount of added RD, the hardness of cooked rice increased, while its resilience and cohesiveness decreased. The addition of 7% RD resulted in maximal adhesive force, gumminess, and chewiness in cooked rice.

Huang et al. [15] reported that 3–5% RD addition increased the hardness and chewiness parameters of a bread prepared from medium-gluten wheat flour, while 1–10% RD addition increased the bread’s springiness, while the cohesiveness was reduced by 3–10% RD addition. In the present study, 3–10% RD addition increased the hardness, chewiness, and springiness of cooked rice but decreased its cohesiveness. These results might suggest that RD addition improves the formation of gluten networks in wheat bread and the kernel texture (mouthfeel) of cooked rice.

### 2.3. Effect of RD Levels on the Tasting Score Evaluation of Cooked White Rice

When compared to cooked IP44 rice, cooked RXY rice had a superior smell, structural appearance, taste, palatability, cool rice texture, and total evaluation score (Table 3). When further compared to no RD addition, both BL addition and LGT addition increased the cooked rice smell, structural appearance, taste, palatability, cool rice texture, and total score. BL-added rice had higher palatability and a higher total score than LGT-added rice. With increases in the amount of added RD, the cooked rice smell, structural appearance, taste, palatability, cool rice texture, and total score all increased.

Chewiness is an important textural parameter for gummies and jellies and plays a key role in establishing elasticity [19]. Kuzey et al. [6] reported that 3.4–9.4% RD addition did not change the hardness and chewiness of extruded soft candy, while 10.7–19.4% significantly increased these two parameters. The authors also reported that 3.4–19.4% RD addition did not change the sensory parameters, such as taste, structure, and general acceptability, of extruded soft candy. The present study found that 3–10% RD addition increased the smell, structural appearance, taste, palatability, and cool rice texture of cooked rice and also increased the hardness, chewiness, adhesiveness, and gumminess. This might be due to RD’s low molecular mass and amorphous phase structure, allowing its free hydroxyl groups to more strongly bind with water molecules. This would enhance the textural firmness of cooked rice by expanding the proportion of soluble solids.

### 2.4. Effects of Adding RD on the Pasting Characteristics of Two Varieties of Rice

The rapid viscosity analysis (RVA) showed that, compared with IP44 flour water suspension, RXY flour water suspension displayed much lower trough, peak, final, and setback viscosity values, as well as pasting temperature and peak time, but exhibited a higher breakdown viscosity (Table 4; Figure S1). Compared with no RD addition, BL and LGT addition significantly lessened the trough, peak, final, breakdown, and setback viscosities but significantly raised the pasting temperature and peak time. With increasing PD concentrations, the peak, trough, breakdown, final, and setback viscosities all decreased significantly, but the peak time and pasting temperature increased.

During the pasting period, starch granules swell, their crystalline regions are disrupted, amylose fractions leach out, and system viscosity increases [20]. Table 4 shows that the two RDs significantly lowered the peak and trough viscosity but increased the pasting temperature, with stronger effects at higher RD levels. These changes might result from the restriction of water availability by RD, which reduces the starch swelling degree and amylose leaching and delays pasting [21,22]. We infer that, in a rice flour water suspension (about 3 g rice flour containing RD/25 g water), free RD molecules can adsorb more water molecules than starch molecules at the pasting stage. Similar findings by Ma et al. [23] suggested that polysaccharides could reduce water availability for the starch pasting process through competitive water molecule binding.

Among the characteristic parameters of a rapid viscosity analysis (RVA), the breakdown viscosity represents the resistance of starch granules to thinning force during continuous heating and mechanical shearing [16]. It is determined as the difference between the peak and trough viscosity, with higher breakdown viscosity indicating much less stable starch granules [21]. The setback viscosity reflects the retrogradation tendency of starch paste during the cooling period, indicating increases in amylose rearrangement and paste viscosity [16]. The setback viscosity of the rice samples significantly decreased with increasing RD addition, suggesting improved system stability. This is possibly because low-viscosity RD molecules form aggregates or coatings on the surface of a starch granule, strengthening the resistance to heat and shear forces [24]. Similarly, the setback viscosity decreased significantly with greater RD addition, likely because of the interactions between RD and leached amylose, which lower the local amylose molecule concentration and disrupt starch recrystallisation, delaying retrogradation [25]. These results are similar to previous studies on the influences of polysaccharides on starch functional properties [26,27].

### 2.5. Effect of RD Levels on the Thermal Properties of Two Varieties of Rice

In terms of the thermal properties measured at day 0, compared with IP44 flour paste, RXY flour paste had a lower enthalpy, *T*_o_, *T*_p_, *T*_c_, and peak height of gelatinisation but a higher gelatinisation peak width (Table 5, Figure S2). When compared to no RD addition, BL and LGT addition significantly reduced the enthalpy value, *T*_c_, and gelatinisation peak width and height but increased *T*_o_; LGT addition increased *T*_p_, but BL addition did not change *T*_p_. With increasing amounts of added PD, the enthalpy, *T*_c_, and gelatinisation peak width and height all decreased, but *T*_o_ and *T*_p_ increased. Further, 3–10% RD addition significantly increased *T*_p_ and decreased the enthalpy of gelatinisation of cooked rice. In contrast, the enthalpy of gelatinisation and *T*_p_ in chiffon cake did not change with 5.24–20.95% and 5.24–15.71% RD addition, respectively, while *T*_p_ was increased with 20.95% RD addition [4]. The differences in findings might be due to the different foods under study.

After the retrograded paste was stored at 4 °C for 21 days, compared with the IP44 paste, the RXY paste had a lower enthalpy, *T*_c_, gelatinisation peak width and height, and amylopectin ageing but higher *T*_o_ and *T*_p_ (Table 6, Figure S3). When further compared with no RD addition, BL and LGT addition decreased the enthalpy value, *T*_c_, and gelatinisation peak width but did not change *T*_o_, gelatinisation peak height, and ageing, and increased *T*_p_. With increasing amounts of added RD, the enthalpy, *T*_o_, *T*_c_, and gelatinisation peak width decreased. The addition of 3–7% RD did not change amylopectin ageing, but 10% significantly increased ageing. The decrease in 21-day measured *T*_c_ with 3–10% RD addition and the maintenance in ageing by 3–7% RD addition suggest that low concentrations of RD can maintain the retrogradation of amylopectin in cooked rice. This result is unsimilar to the effect of polydextrose addition on a cooked japonica-type rice [18].

### 2.6. Influence of Adding RDon the Thermal–Mechanical Properties of Two Varieties of Milled Rice

When compared with IP44 rice dough, RXY had a higher dough development time (DDT), maximum consistency (C1), minimum consistency (C2), and protein weakness degree (C1–Cs), and higher starch breakdown torque (C3–C4), endogenous amylase activity (C3/C4), and heating speed (α) but a lower dough stability time (DST), maximum gelatinisation torque (C3), persistent viscosity (C4), setback end torque (C5), starch setback (C5–C4), and gelatinisation speed (β) (Table 7).

Compared with no RD addition, BL and LGT addition did not change the dough development time and stability time, persistent viscosity, setback end torque, starch setback, and gelatinisation speed but reduced the maximum consistency, minimum consistency, maximum gelatinisation torque, protein weakness degree, starch breakdown, amylase activity, and heating speed. The decrease in the amylase activity of rice dough by 3–10% BL or LGT addition is similar to the reduced invitro hydrolysis rate of corn starch with increasing RD inclusion levels [16]. Reduced setback viscosity was also associated with increased RD addition (Table 4), indicating lower digestibility of endogenous amylase.

With increases in the added RD amount, the dough development time and dough stability, persistent viscosity, setback end torque, starch setback, and gelatinisation rate remained unchanged, but the maximum consistency, minimum consistency, maximum gelatinisation torque, protein weakness degree, starch breakdown, amylase activity, heating speed, and enzymatic degradation speed (γ) were reduced.

DST reflects the resistance force of the dough against the blades and is a key indicator of flour processing quality [15]. Huang et al. [15] showed that 3–10% RD addition increased the development time and stability time of wheat dough but decreased the softening index measured by micro-farinograph analysis. In the present study, 3–10% RD did not change the development time and stability time of rice flour dough but decreased the degree of protein weakness. This means that 3–10% RD addition can enhance new protein aggregation and interface reconstruction in rice flour dough.

### 2.7. Influence of RD Amount on the Starch Crystallinity Degree and Protein Secondary Conformation of Cooked White Rice

Compared with cooked IP44 rice, cooked RSY rice had similar R_1022/995_ and R_1047/1022_ values, higher R_1068/1022_ and beta (β)-sheet values, and fewer random coils, alpha (α)-helixes, and beta(β)-turns (Table 8, Figures S4 and S5). Compared with no added RD, both BL and LGT significantly increased R_1022/995_ but did not significantly change R_1047/1022_, R_1068/1022_, β-sheets, and α-helixes. BL addition increased random coils, but LGT decreased β-turns. The addition of 3–10% and 5–7% RD significantly increased R_1022/995_ and R_1047/1022_, respectively. The addition of 10% RD resulted in fewer β-sheets and more random coils and α-helixes relative to no RD addition. Combined with the decrease in C2 torque and protein weakness degree (C1–Cs) in rice dough, these results suggest that RD addition might increase the amorphous and crystalline regions of starch in cooked rice, consistent with the formation of new gel-like structures. A total of 10% BL or LGT significantly induced the formation of α-helix and random coil secondary protein structures.

### 2.8. Main Effect of Rice Cultivar, RD Species, and Addition Levels on the Cooking of White Rice with Principal Component Analysis (PCA) and Scree-Plot

**Figure 1:**
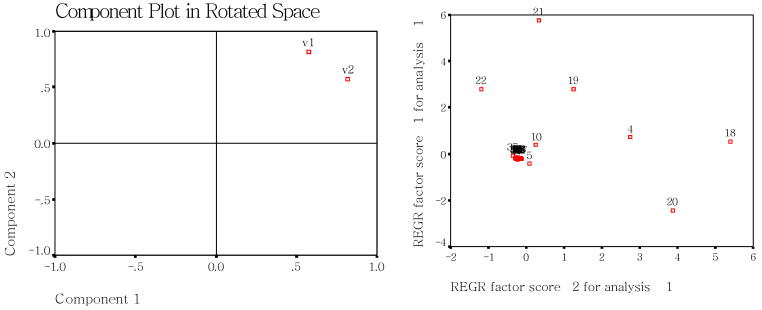
Main effect analysis of rice varieties on the 59 parameters determined in this study via PCA analysis. Notes: v1, RXY rice variety; v2, IP44 rice variety; 1, Cooking time (min); 2, water absorption ratio; 3, Gruel solid loss (mg/g); 4, Hardness; 5, Adhesive force (g); 6, Adhesiveness (mJ); 7, Resilience (0.01); 8, Cohesiveness (0.01); 9, Springiness (mm); 10, Gumminess (g); 11, Chewiness (mJ); 12, Smell value; 13, Appearance structure of cooked rice; 14, Palatability value; 15, Taste value; 16, Cool rice texture; 17, Total sensory score; 18, RVA peak viscosity; 19, RVA trough viscosity; 20, RVA breakdown viscosity; 21, RVA final viscosity; 22, RVA Setback viscosity; 23, RVA Pasting peak time; 24, RVA Pasting temperature; 25, Gelatinisation enthalpy-0 d; 26, T_o_-0 d; 27, T_p_-0 d; 28, T_c_-0 d; 29, Peak width-0 d; 30, Peak height-0 d; 31, Enthalpy-21 d; 32, T_o_-21 d; 33,T_p_-21 d; 34, T_c_-21 d; 35, Peak width-21 d; 36, Peak height-21 d; 37, Ageing; 38, DDT; 39, DST; 40, C1; 41, Cs; 42, C2; 43, C3; 44, C4; 45, C5; 46, C1–Cs torque; 47, C3–C4 torque; 48, C3/C4; 49, C5–C4 torque; 50, α (heating rate); 51, β (gelatinisation rate); 52, γ (enzymatic degradation rate); 53, R_1022/995_; 54, R_1047/1022_; 55, R_1068/1022_; 56, beta-sheets; 57, random coils; 58, alpha-helixes; 59, beta-turns.

Algebraically, principal component analysis (PCA) involves performing eigenvalue decomposition on the covariance matrix of standardised data, where eigenvectors form a new coordinate system, and the eigenvalues represent the variance magnitude of the corresponding principal components [17]. In the present study, the average values of 59 parameters from seven experiments (Figure 1, notes) were used for PCA analysis to evaluate the influence of rice variety, RD species, and addition amount on cooking rice. Figure 1 is the loading scatter plot of the principal components in rotated space after varimax rotation; the two rice cultivars were positively loaded and clearly separated, indicating a significant main effect of rice cultivar on the physicochemical, thermal, and functional quality parameters of rice. A factional scatterplot of the principal components of 59 determined parameters showed that hardness (4), adhesive force (5), gumminess (10), RVA peak viscosity (18), RVA trough viscosity (19), RVA breakdown viscosity (20), RVA final viscosity (21), and RVA setback viscosity (22) exhibited scattered distributions, while the other 51 parameters were amassed together. In principal component 1 (PC1), the maximum score was RVA final viscosity (21), and the minimum score was RVA breakdown viscosity (20). RVA final viscosity (21), RVA setback viscosity (22), RVA trough viscosity (19), RVA peak viscosity (18), hardness (4), and gumminess (10) were located at the left end of the horizontal axis, indicating greater effects on rice cooking quality than adhesive force (5) and RVA breakdown viscosity (20), located at the right end of the horizontal axis. In PC2, the maximum score was RVA peak viscosity (18), and the minimum score was RVA setback viscosity (22). RVA peak viscosity (18), RVA breakdown viscosity (20), hardness (4), RVA trough viscosity (19), RVA final viscosity (21),gumminess (10), and adhesive force (5) were located at the right end of the horizontal axis, indicating greater effects on rice cooking quality than RVA setback viscosity (22), located at the left end of the horizontal axis. These results show that the above eight parameters are very sensitive indices for evaluating the quality of the two rice cultivars when being cooked.

**Figure 2:**
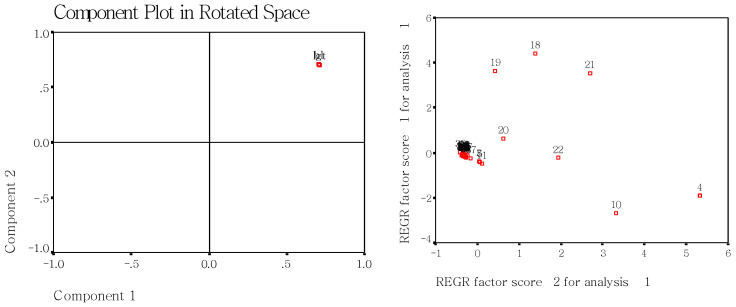
Main effect of RD species on the 59 parameters determined in this study using PCA analysis. Notes: lb, LB; lgt, LGT; 1–59 are the same as Figure 1.

Figure 2 shows the influence of RD species on thedetermined 59 parameters of cooked rice. The effects of RD species (LB and LGT) were similar and positively loaded. In PC1, the maximum score was RVA peak viscosity (18), and the minimum score was gumminess (10). RVA final viscosity (21), RVA peak viscosity (18), RVA trough viscosity (19), and RVA breakdown viscosity (20) were located at the left end of the horizontal axis, indicating greater effects on rice cooking quality than hardness (4) and gumminess (10), located at the right end of the horizontal axis.

In PC2, the maximum score was hardness (4), and the minimum score was resilience value (7). Hardness (4), gumminess (10), RVA final viscosity (21), RVA setback viscosity (22), RVA peak viscosity (18), RVA breakdown viscosity (20), RVA trough viscosity (19), and chewiness (11) were located at the right end of the horizontal axis, indicating greater effects on rice cooking quality than the resilience value (7), located at the left end of the horizontal axis. Ten parameters sensitive to RD addition species in cooking rice were gruel solid loss of cooking process (3), hardness value (4), resilience value (7), gumminess value (10), chewiness value (11), RVA trough viscosity (19), peak viscosity (18), final viscosity (21), breakdown viscosity (20), and setback viscosity (22).

**Figure 3:**
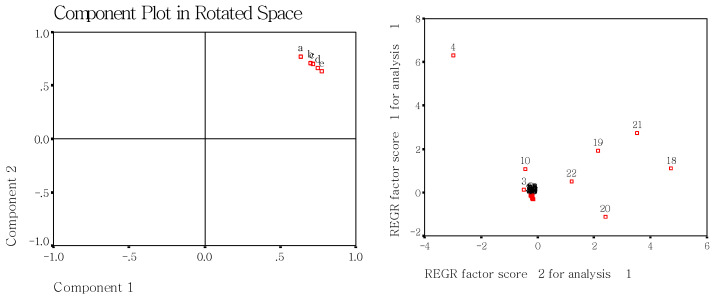
Main effect of RD addition amount on the 59 parameters determined in this study using the PCA method. Notes: a,0% RD level; b, 3% RD level; c, 5% RD level; d, 7% RD level; e, 10% RD level; 1–59 are the same as Figure 1.

Figure 3 shows the main effect of the RD addition amount on the 59 determined parameters of cooked rice. The effect of the RD addition amount (0, 3, 5, 7, and 10%) was consistent and positively loaded. In PC1, the maximum score was hardness (4), and the minimum score was RVA breakdown viscosity (20). Hardness (4), RVA peak viscosity (18), RVA final viscosity (21), RVA trough viscosity(19), RVA setback viscosity (22), and gumminess (10) were located at the left end of the horizontal axis, indicating greater effects on rice cooking quality than RVA breakdown viscosity (20), located at the right end of the horizontal axis.

In PC2, the maximum score was RVA peak viscosity (18), and the minimum score was hardness (4). RVA peak viscosity (18), RVA final viscosity (21), RVA trough viscosity (19), breakdown viscosity (20), and setback viscosity (22) were located at the right end of the horizontal axis, indicating greater effects on rice cooking quality than gumminess (10) and hardness (4), located at the left end of the horizontal axis.

The eight sensitive parameters to RD addition amount in cooking rice included gruel solid loss of the cooking process (3), hardness value (4), gumminess value (10), trough viscosity (19), peak viscosity (18), final viscosity (21), breakdown viscosity (20), and setback viscosity (22).

Freshness detection based on pH changes of brown rice or milled rice has a low accuracy rate due to the vast number of varieties and types of rice. Thus, rice breeding experts and storage experts have been searching for an improved freshness detection method for rice [28]. The present study used the addition of RD in cooking rice and PCA to show that RD addition during rice cooking significantly influenced the cooking, textural, and pasting properties. These results are similar to those of two early indica rice varieties in a 12-month storage experiment [17].

### 2.9. Effect of RD on the Microstructural Changes of Cooked Rice

**Figure 4:**
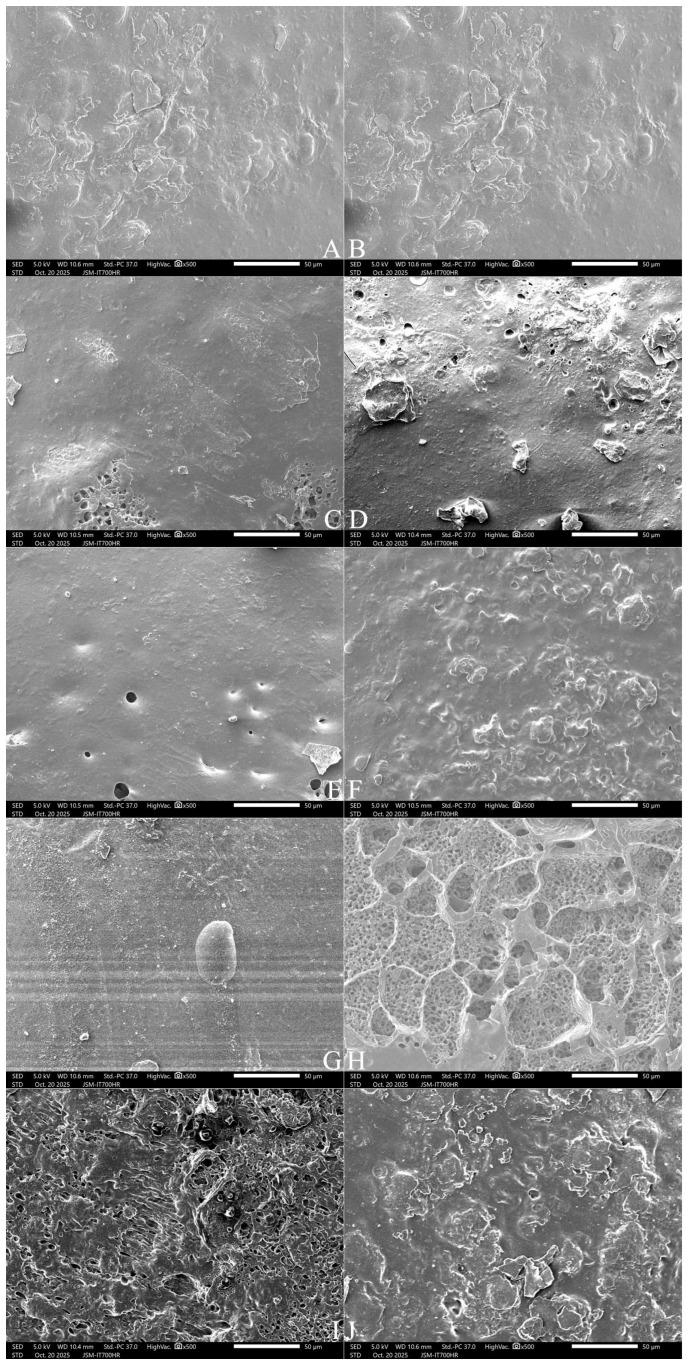
Modified effect of BL or LGT on the surface structure of cooked RXY white rice. Notes: (**A**,**B**) are the controls; (**C**,**E**,**G**,**I**) are the samples with added 3%, 5%, 7%, and 10% BL, respectively; (**D**,**F**,**H**,**J**) are the samples with added 3%, 5%, 7%, and 10% LGT, respectively. All the photos are magnified 500×.

**Figure 5:**
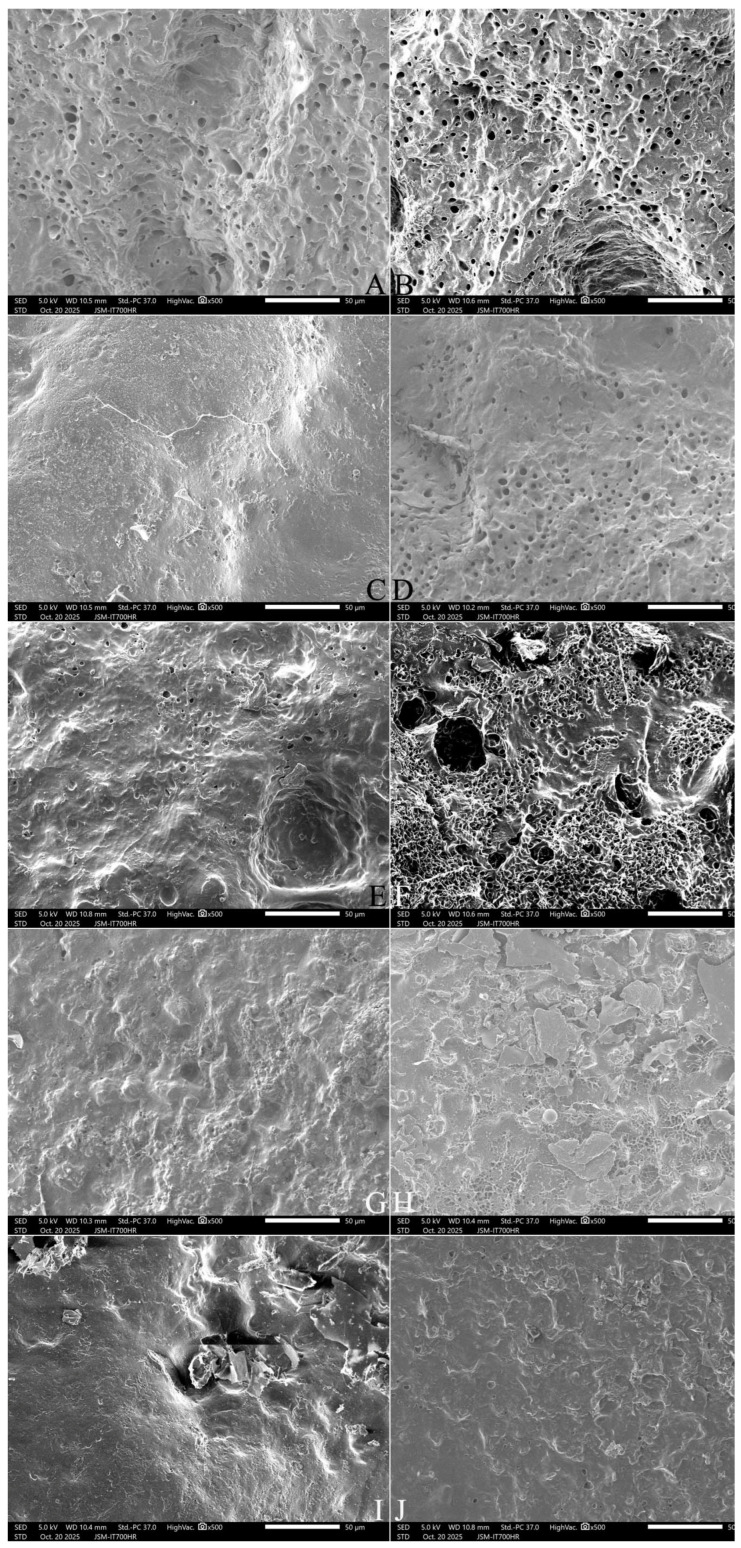
Modified effect of BL or LGT on the surface structure of cooked IP44 white rice. Notes: (**A**,**B**) are the controls; (**C**,**E**,**G**,**I**) are the samples with added 3%, 5%, 7%, and 10% BL, respectively; (**D**,**F**,**H**,**J**) are the samples with added 3%, 5%, 7%, and 10% LGT, respectively. All the photos are magnified 500×.

The surface of cooked RXY rice with no BL or LGT addition was relatively smooth (Figure 4A,B). Compared to the control sample, 3% RD addition resulted in larger, sparser pores on the kernel surface (Figure 4C,D), which were formed due to the escape of water molecules when the samples were freeze-dried. The addition of 5% BL resulted in much larger pores (Figure 4E). The addition of 7–10% LGT also resulted in filamentous zoning structures with innumerable small pores (Figure 4H and Figure 5I).

Cooked IP44 rice with no BL or LGT addition exhibited small pores and large, deep holes in the surface (Figure 5A,B). The addition of 3–10% BL resulted in fewer and shallower pores and an uneven surface (Figure 5C,E,G,I). The addition of 3% LGT resulted in an undulated surface with many pores (5D). The addition of 5% LGT resulted in an uneven surface with a few round shallow holes (5F), and 7% LGT addition resulted in a lamellar stacking surface (5H). The stacked lamellas were fused into gel structures with micropores (5J).

**Figure 6:**
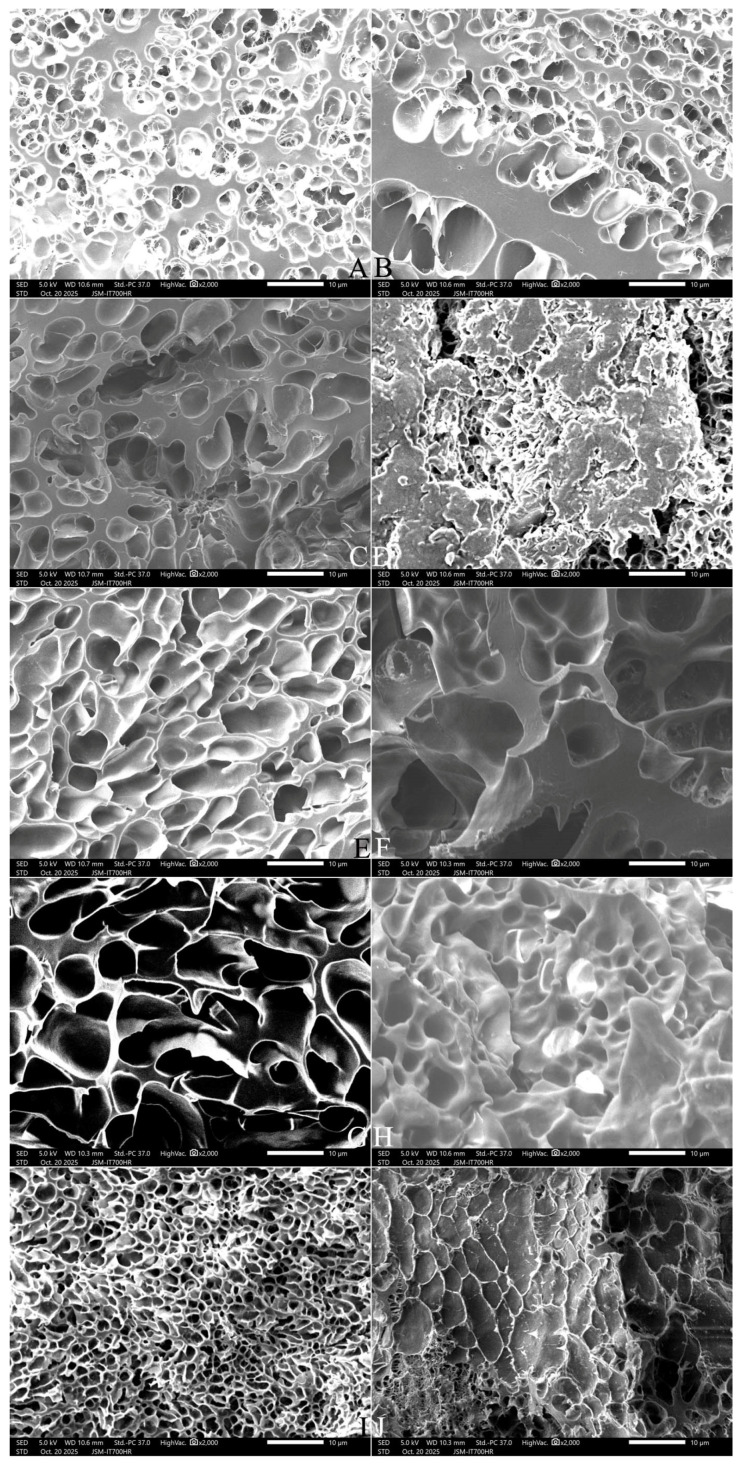
Effect of BL or LGT on the transverse-section structure of cooked RXY white rice. Notes: (**A**,**B**) are the controls; (**C**,**E**,**G**,**I**) are the samples with added 3%, 5%, 7%, and 10% BL, respectively; (**D**,**F**,**H**,**J**) are the samples with added 3%, 5%, 7%, and 10% LGT, respectively. All the photos are magnified 2000×.

**Figure 7:**
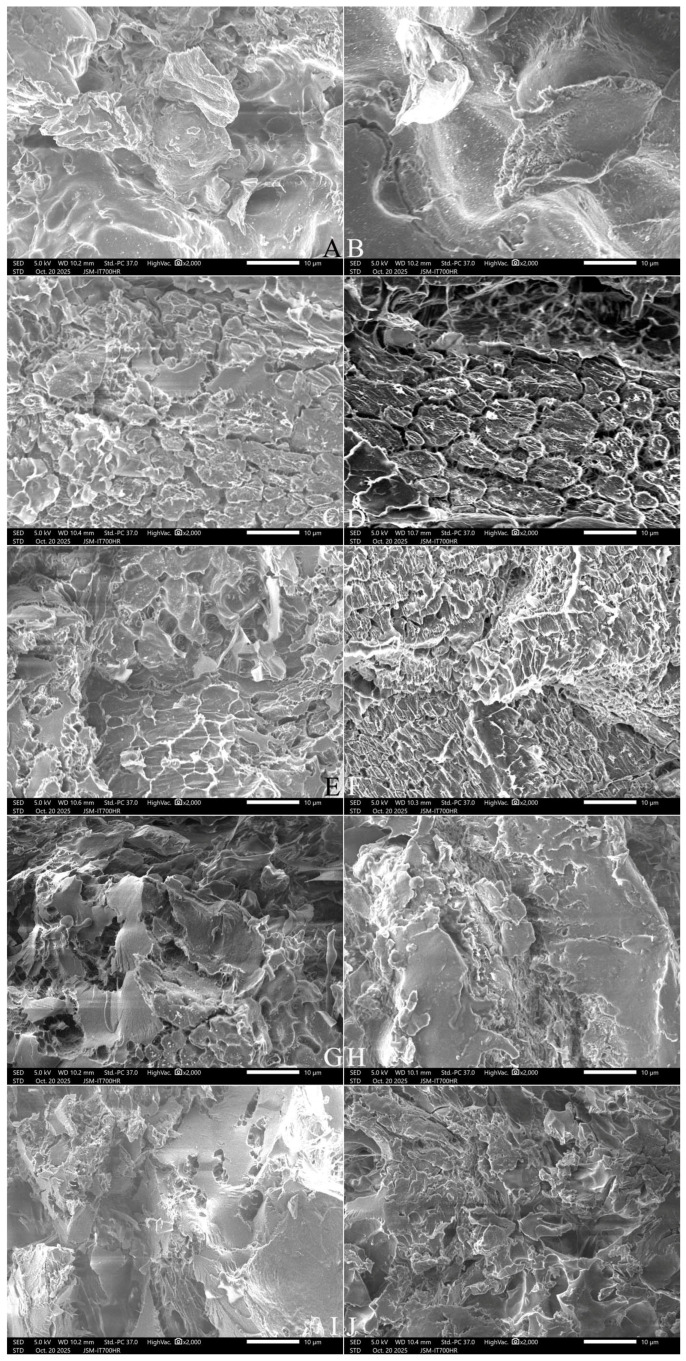
Modification of BL or LGT on the transverse-section structure of cooked IP44 white rice. Notes: (**A**,**B**) are the controls; (**C**,**E**,**G**,**I**) are the samples with added 3%, 5%, 7%, and 10% BL, respectively; (**D**,**F**,**H**,**J**) are the samples with added 3%, 5%, 7%, and 10% LGT, respectively. All the photos are magnified 2000×.

Cooked rice kernels exhibited cracks in the cross-section because of the entry of water molecules, and the japonica RXY variety (Figure 6A,B) had smaller, shallower cracks and more filament aggregates than the early indica IP44 variety (Figure 7A,B). The IP44 rice had much narrower and deeper cracks that extended directly into the deep endosperm tissue (Figure 7A,B). The addition of BL and LGT during the cooking of RXY rice caused increases in the matrix wall thickness and a gradual increment in the open pore size and depth (Figure 6C–H). The addition of 10% BL (Figure 6I) and LGT (Figure 6J) induced innumerable filamentous and thin lamellar gel-like structures in the cross-section. These results suggest that more elastic gel networks form in cooked rice at high concentrations of RD.

With increasing BL and LGT addition during the cooking of indica IP44 rice, the cross-linked gel-like structures gradually became more pronounced (Figure 7C–J). These results suggest that the admission of water molecules into a rice kernel is promoted by BL and LGT, which exacerbates the swelling extent of starch granules, making their internal structure loose and creating gel-like structures in the centre area of the cross-section. The new gel-like structures may be due to the low viscosity and high adhesiveness of BL and LGT in aqueous solution [15]. In the present cooking test, the ratio of rice sample to cooking water was 1:1.3; RD molecules could cover the surface of a rice kernel, and water molecules could be easily transferred into the rice kernel via their strong hygroscopic property.

**Figure 8:**
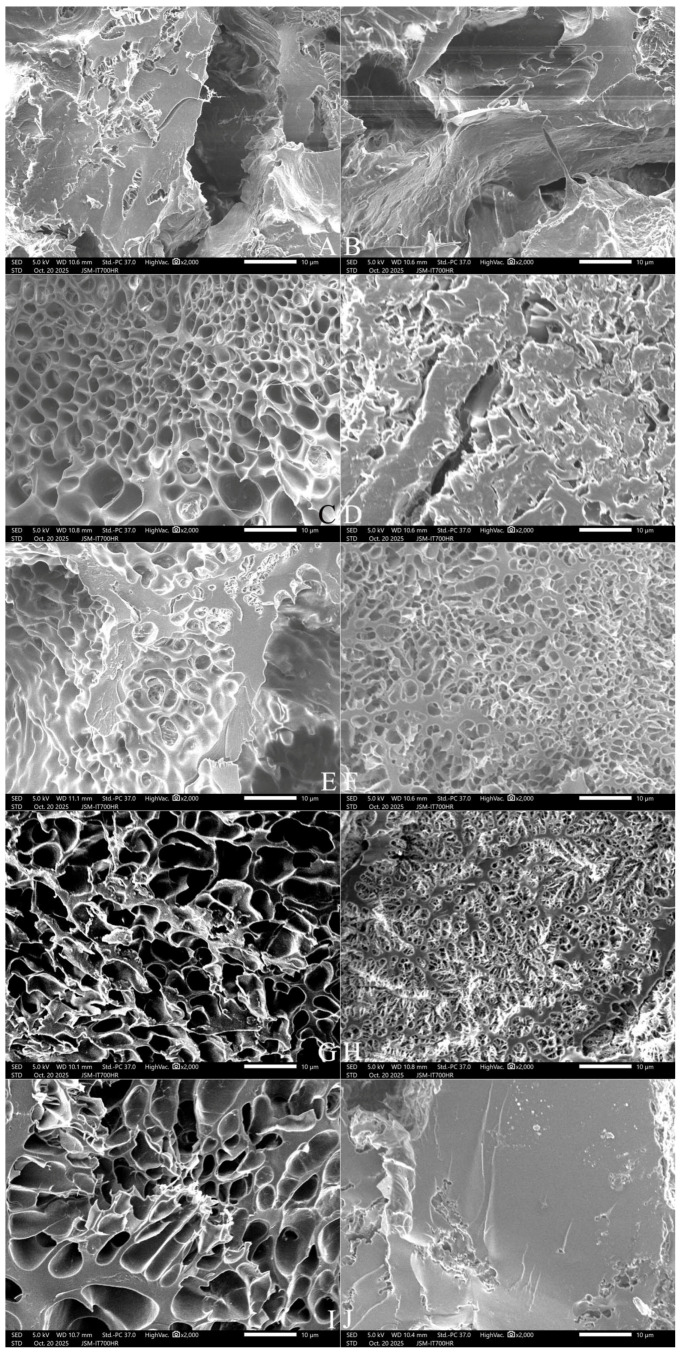
Effect of BL or LGT on the lengthwise-direction section structure of cooked RXY white rice. Notes: (**A**,**B**) are the controls; (**C**,**E**,**G**,**I**) are the samples with added 3%, 5%, 7%, and 10% BL, respectively; (**D**,**F**,**H**,**J**) are the samples with added 3%, 5%, 7%, and 10% LGT, respectively. All the photos are magnified 2000×.

**Figure 9:**
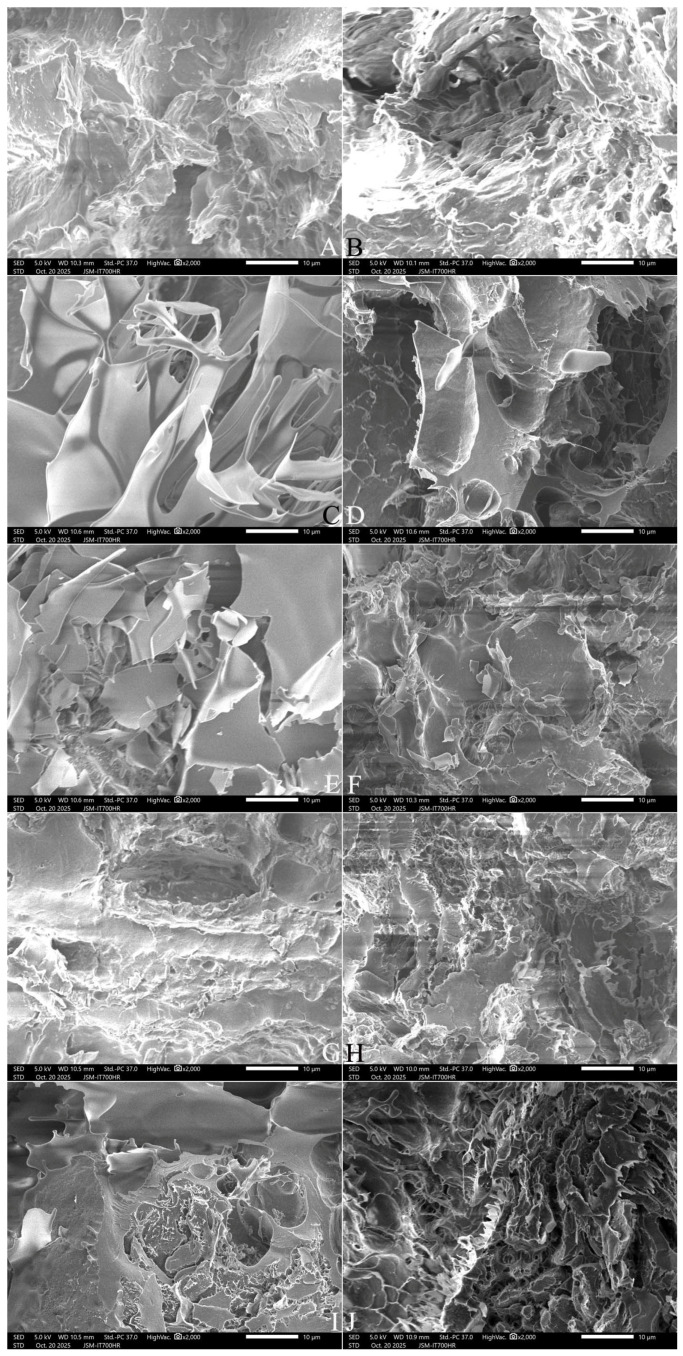
Effect of BL or LGT on the lengthwise-direction section structure of cooked IP44 white rice. Notes: (**A**,**B**) are the controls; (**C**,**E**,**G**,**I**) are the samples with added 3%, 5%, 7%, and 10% BL, respectively; (**D**,**F**,**H**,**J**) are the samples with added 3%, 5%, 7%, and 10% LGT, respectively. All the photos are magnified 2000×.

Figure 8 and Figure 9 show that the cooked RXY (Figure 8A,B) and IP44 rice (Figure 9A,B) had larger pores and much thicker starch bodies in the longitudinal section. An increase in the amount of added BL or LGT, from 3% to 7%, resulted in more pores and filaments in RXY rice (Figure 8C–H). With the addition of 10% BL, RXY rice exhibited deeper and thicker-walled honeycomb structures (Figure 8I). The addition of 10% LGT resulted in gel-like starch bodies with many interspersed pores (Figure 8J).

With an increase in the amount of added BL or LGT, from 3% to 5%, striped structures with large and deep holes formed in IP44 rice (Figure 9C–F). With the addition of 7% and 10% BL or LGT, despite the large holes, the gel network became relatively continuous, indicating the formation of a new gel network (Figure 9G–J).

The addition of RD has different effects on the hardness of baked foods. Miyazaki et al. [29] and Yu et al. [7] reported that 2.5–7.5% RD and 8.05% RD addition increased the hardness of bread and biscuits, respectively, while Lin and Lee [4] reported that 5.24–15.71% RD addition did not change the hardness of chiffon cake and 20.95% RD addition significantly increased the hardness. Huang et al. [15] found that, compared with the no RD addition, 3–5% RD addition increased the hardness and chewiness texture of bread, but 7–10% RD addition decreased it. Further, 3–10% RD addition decreased the bread soft index and cohesiveness. In the present study, 3–10% RD addition increased the hardness, chewiness, and springiness of cooked rice. We speculate that the hygroscopic RD particles might be firmly bonded to water molecules, reducing the usable water molecules for relatively sufficient starch gelatinisation in the baking and cooking process and resulting in a higher hardness texture; the more elastic gel network structures in dough or cooked rice are formed by RD after hydration.

Because polydextrose products have undesirable colour and flavour characteristics, RD, as a starch-based, low-calorie bulking agent, has been explored [30]. In our recent study [18], the addition of 3–10%polydextrose indeed significantly reduced the hardness, chewiness, and springiness of cooked japonica rice. This might be due to the stronger hygroscopicity of polydextrose, with an equilibrium moisture content of 69.43% (adsorption) and 64.09% wet basis (desorption) at 0.98 water activity and 37 °C [31]. The equilibrium moisture content of RD remains to be determined.

Here, particular attention was given to torque C1 (maximum torque during kneading), which is evidently affected by the number of water molecules taken in by a rice flour mix [32]. Increasing the addition amount of RD powder (0–10%) indeed markedly reduced the torque C1 of a Mixolab measurement. This shows a reduced initial protein structuring capacity (kneading phase). C2 torque (an indicator of the protein weakening degree because of mechanical/thermal stress) and C1–Cs were also significantly decreased with increasing RD level. This is because RD induces a weakening process in the protein network under the coupled action of shearing plus temperature elevation [33]. This result is similar to the reduction in C2 torque in 5–20% chestnut flour-fortified wheat dough [32]. This reduction can be attributed to the dilution of glutelin and the interference of added fibres in rice dough.

Regarding starch, analysis of C3 (starch gelatinisation) revealed a gradual reduction in gelatinisation intensity, while C4 (thermal stability of starch gel) did not change as the amount of added RD increased. Indeed, the decrease in C3 reflects poor retention of water molecules and inefficient structuring of the starch network. This finding is consistent with a previous report [34], where adding fibre-rich ingredients modified water accessibility and induced the emergence of more cohesive starch hydrogels. The level of C4 torque remained unchanged when increasing the amount of added RD, suggesting no change in the heat resistance force of the rice dough. C5 torque indicates the starch setback degree after the cooling process, and its reduction can be beneficial in light of the preservation of finished foods because it can limit the staling degree of bakery products [35]. In the present study, C5 and setback torque (C5–C4) remained unchanged with increasing RD addition, indicating unchanged starch retrogradation in rice dough after cooling. Thus, the incorporation of 3–10% RD during rice cooking weakened the protein structure and reduced starch gelatinisation but did not change the gel thermal stability and post-cooling retrogradation of rice dough, thereby improving the dietary fibre content and chewiness of cooked rice. These results highlight the relative importance of adjusting RD proportions during rice cooking to balance the sensory, nutritional, and technological stability properties of rice.

## 3. Conclusions

This study is the first to add RD in cooking rice and then analyse the functional, thermal–mechanical, and structural properties of the cooked rice. RD provides water retention, low viscosity, and heat/acid stability due to its molecular structure and hydrophilic groups; these properties reduced the rice cooking time, enthalpy and conclusion temperature of gelatinisation, and peak and setback viscosity and increased the gruel solid loss, peak temperature of gelatinisation and pasting, crystalline and amorphous regions of starch, and new gel-like structures in cooked rice, giving rise to cooked rice that was harder, chewier, and more adhesive, with an elastic texture. With increases in the amount of added RD, the rice dough development time and stability, persistent viscosity, setback end torque, starch setback viscosity, and gelatinisation rate remained unchanged, but the maximum consistency, minimum consistency, maximum gelatinisation torque, protein weakness degree, starch breakdown, amylase activity, heating speed, and enzymatic degradation speed were reduced. Both BL addition and LGT addition increased the smell, structural appearance, palatability, taste, cool rice texture, and total score of cooked rice. Principal component analysis and factor analysis showed that the sensitive parameters for RD addition in cooking rice included cooking properties (gruel solid loss), textural parameters (hardness, resilience, gumminess, chewiness), and pasting parameters (trough viscosity, peak viscosity, final viscosity, breakdown viscosity, and setback viscosity).Future studies will need to determine the hygroscopicity of RD and its molecular structure, as well as the palatability/health mechanism of cooked rice with added RD.

## 4. Materials and Methods

### 4.1. Milled Rice Samples

A sample of the “Ruan Xiangyu” (RXY) variety of japonica paddy rice was collected from the Zhangjiagang Grain Storage Depot, Jiangsu Province, China. The early indica rough rice of the IP44 variety was obtained from the Zhongshan Grain Storage Depot, Guangdong Province, China. The two paddy samples had a wet basis moisture content (MC) of 13.37% and 12.82%, respectively (Table 9). The Bailong (BL, Biolong Biotechnology Company, Dezhou city, China; Chinese product) and Luogai Te (LGT, Rogette Biological Nutritional Products (Wuhan, China) Co., Ltd., French product) samples of resistant dextrin, with respective MCs of 6.9% and 8.0%, were obtained from the Anhui Runloy Biotechnology Company, Huaibei city, China. The two paddy samples were milled into white rice using a rice polisher, and the white rice was then milled into rice flour with a high-rate pulveriser. BL and LGT powders at weight ratios of 0%, 3%, 5%, 7%, and 10%were added to the two varieties of white rice grains or flour to obtain the test samples.

The sample MCs in Table 9 were obtained by oven drying in accordance with the method of AOAC [36]. The grain length-to-width ratio was obtained with an appearance quality rapid-scanning device for milled rice [18]. The contents of amylose and protein in milled rice were measured using the GB/T 15683—2008 method [37] and ISO 14891—2002 [38], respectively. The free fatty acid (FA) content of rice flour was determined using the GB/T 5510–2024 standard [39].

### 4.2. The Optimal Cooking Time and Gruel Solid Loss

The measurement method for the optimal cooking time of rice kernels at normal atmospheric pressure was performed according to Liu et al. [18]. Two grams of white rice sample was immersed for 5.1 min in 20 mL deionized water in a round-bottom glass tube and then steamed in a boiling water pan. During the cooking process, a single rice kernel was extracted at 30.0 s gapand pressed on a robust glass plate with the bottom of a spoon head, and the optimal cooking time was obtained when the white core in the kernel was not observed when pressed. Based upon the optimal cooking time, the gruel solid loss and water uptake ratio in the cooking test were obtained according to the protocol of Liu et al. [18].

### 4.3. Textural Characteristics of Cooked White Rice

The textural profile of cooked milled rice was tested using a textural profile device (CTX, Brookfield, Middleboro, MA, USA). The rice was steamed according to the GB/T 15682—2008 protocol [40]. BL or LGT was added to the rice kernel samples at a mass ratio of 0% to 10%, with a total mass of 15 g for rice kernels plus the RD powder. The ratio of the sample to cooking water was set at 1:1.30. Samples were immersed for 30.0 min at room temperature (RT) and, afterward, steamed for 40 min in a pan. After being braised for 20 min, the sample measurements were performed using the P35 cylindrical probe of the textural device. The adopted parameters were as follows: the pre-test, test, and post-test speeds were 2 mm/s; the compression distance was set at15.0 mm. The textural parameters included hardness, adhesive force, adhesiveness, cohesiveness, resilience, springiness, gumminess, and chewiness. Springiness sensorily is the height to which the food recovers between the first and second bites. Gumminess (hardness × cohesiveness, g) is the energy needed to break a semisolid sample into a stable status fit for swallowing. Chewiness (hardness × cohesiveness × springiness, mJ) is the energy needed to chew a solid sample into a stable status fit for swallowing.

### 4.4. Pasting Profile Parameters

A rapid viscosity device (RVA—TecMaster, Rui-hua Perten Scientific Device Co., Beijing, China) was employed to measure the mixtures of rice flour and RD powder using the GB/T24852—2010 standard [41]. The pasting parameters included the peak, trough, breakdown, final, and setback viscosities, as well as the peak time and pasting temperature.

### 4.5. Thermal Characteristics

Rice flour and BL or GLT powder were well mixed, and then aliquots of the sample were weighed for gelatinisation property analysis in a differential scanning calorimeter (DSC 214.0, Netzsch GmbH, Freistaat Bayern, Germany) using the approach of Liu et al. [18]. After gelatinisation, the sample pans were kept at 4 °C for three weeks and then determined for the setback process. Equation (1) was used to calculate amylopectin ageing in retrograded flour paste:

Ageing (%) = (Enthalpyof gelatinisation at 21-day)/(Enthalpy of gelatinisation at 0-day) × 100(1)

### 4.6. Thermo-Mechanical Characteristics

A Mixolab (Chopin Technologies Co., Tripette et Renaud, Paris, France) was used to analyse the thermal–mechanical curves of rice flour dough, as described by Wang et al. [42]. The assays were executed at a fixed water hydration rate of 60%.

### 4.7. Fourier Transform Infrared Spectroscopy (FTIR)

The freeze-dried specimens of cooked rice were measured on a Nicolet 6700 FTIR device (Thermo Fisher Scientific, Waltham, MA, USA) for starch crystallinity and protein secondary conformation using the protocol of Liu et al. [18]. Frozen samples of cooked rice were lyophilised to about 1.6% wet basis moisture content. An aliquot of the sample was pestled for 2 min with potassium bromide powder at a ratio of 1:100 and then processed into tablets. The test conditions were as follows: the wavenumber of scanning was in the range of 400–4000 cm^−1^; the spectral resolution was 4 cm^−1^ with a 100% T-line signal-to-noise ratio of 4300 to 4400 cm^−1^, 64 scans. Each sample was scanned in three different regions. The wavenumber ratio R_1068/1022_ indicates the ratio value of protein to starch; R_1047/1022_ and R_1022/995_ reflect the short extent of a starch granule surface, indicating the crystalline zones and amorphous zones of starch, respectively [43]. The second-level protein structures determined in an amide I region included: α-helixes (1650 to 1660 cm^−1^), β-turns (1660 to 1700 cm^−1^), β-sheets (1600 to 1640 cm^−1^), and random coils (1640 to 1650 cm^−1^).

### 4.8. Sensory Analysis of Cooked Rice

The cooked rice was sensorily assessed using the GB/T 15682―2008 method [40]. Eight trained evaluators (4 females and 4 males) aged 23–26 years observed, tasted, ate, and assessed the cooked rice. The trained evaluators could not smoke or eat food one hour before the tasting test, but they were allowed to drink water.

### 4.9. Scanning Electron Microscopy (SEM)

The cooked rice samples were frozen in a −20 °C refrigerator and then lyophilised in a freeze dryer before SEM observation. The freeze-dried samples were gold-sprayed and then photographed on a scanning electron microscope according to the procedure of Wang et al. [43]. Each sample was carefully observed and photographed at 100 to 3000 times magnification under 25kV accelerating voltage.

### 4.10. Data Analysis

A randomised block design was employed to investigate the effects of rice variety, resistant starch species, and addition level on the physicochemical and quality indices of cooked rice. Except for the sensory evaluation of cooked rice, which was repeated eight times, all other experiments were repeated three times. The data were examined using SPSS (Version 17.0, SPSS Incorporated Company, Armonk, NY, USA) [44]. The quality indices of the two samples of raw milled rice were compared during *t*-tests. To examine the main effects of rice variety, RD species, and addition level, UGLM analysis was used; multiple comparisons and the LSD test were adopted to compare the significance of the means. For the UGLM analysis, each rice variety, each resistant starch species, and each addition level separately had 80, 80, and 32 data points for sensory evaluation, but 30, 30, and 12 data points for the other trials. Significance was fixed at *p* < 0.05. The data reduction method and factor analysis were adopted for principal component analysis (PCA) and to obtain the factional scatter-plot.

## Figures and Tables

**Table 1:** Effect of adding RD on the rice cooking process.

Factor	Levels	Optimal Cooking Time (min)	Water Absorption Ratio	Gruel Solid Loss (mg/g)
Rice	RXY	18.62 ± 0.07 ^g^	2.99 ± 0.04 ^d^	83.59 ± 1.59 ^c^
variety	IP44	21.52 ± 0.07 ^a^	3.16 ± 0.04 ^a^	75.58 ± 1.59 ^e^
RD	BL	20.33 ± 0.07 ^c^	3.05 ± 0.04 ^b^	82.04 ± 1.59 ^cd^
species	LGT	19.80 ± 0.07 ^d^	3.10 ± 0.04 ^abc^	77.13 ± 1.59 ^e^
Addition	0	21.06 ± 0.12 ^b^	3.21 ± 0.07 ^a^	45.25 ± 2.52 ^g^
(%)	3	20.39 ± 0.12 ^c^	2.95 ± 0.07 ^d^	67.22 ± 2.52 ^f^
	5	19.98 ± 0.12 ^d^	3.15 ± 0.07 ^ab^	79.00 ± 2.52 ^de^
	7	19.66 ± 0.12 ^e^	3.09 ± 0.07 ^ab^	91.66 ± 2.52 ^b^
	10	19.24 ± 0.12 ^f^	2.99 ± 0.07 ^cd^	114.81 ± 2.52 ^a^

Notes: RD, resistant dextrin; RXY, Ruan Xiangyu; All data are shown as mean ± SD; number of trials―n = 3, and each rice variety, each RD species, and each RD addition level separately have 30, 30, and 12 data points for UGLM analysis. Different superscript letters represent significant differences (*p* < 0.05) within the column.

**Table 2:** Effect of RD on the textural characteristics of two varieties of cooked white rice.

Factor	Levels	Hardness (g)	Adhesive Force (g)	Adhesiveness (mJ)	Resilience (0.01)	Cohesiveness (0.01)	Springiness (mm)	Gumminess (g)	Chewiness (mJ)
Rice	RXY	2100 ± 25 ^e^	121.5 ± 2.6 ^a^	2.99 ± 0.11 ^a^	10.6 ± 0.2 ^d^	25.8 ± 0.3 ^d^	9.28 ± 0.18 ^abc^	539 ± 10 ^e^	49.2 ± 1.8 ^e^
variety	IP44	2373 ± 25 ^b^	26.6 ± 2.6 ^g^	0.60 ± 0.11 ^f^	14.5 ± 0.2 ^a^	31.9 ± 0.3 ^a^	9.26 ± 0.18 ^abc^	747 ± 10 ^a^	68.9 ± 1.8 ^a^
RD	BL	2272 ± 25 ^c^	76.6 ± 2.6 ^cd^	1.92 ± 0.11 ^bc^	12.6 ± 0.2 ^bc^	29.5 ± 0.3 ^b^	9.34 ± 0.18 ^abc^	667 ± 10 ^c^	62.0 ± 1.8 ^bc^
species	LGT	2202 ± 25 ^d^	71.3 ± 2.6 ^e^	1.67 ± 0.11 ^d^	12.5 ± 0.2 ^bc^	28.2 ± 0.3 ^c^	9.19 ± 0.18 ^bc^	619 ± 10 ^d^	56.1 ± 1.8 ^d^
Addition	0	1754 ± 39 ^f^	58.1 ± 4.1 ^f^	1.34 ± 0.18 ^e^	14.3 ± 0.3 ^a^	32.2 ± 0.5 ^a^	8.54 ± 0.28 ^d^	560 ± 16 ^e^	46.8 ± 2.7 ^e^
	3	2277 ± 39 ^c^	82.5 ± 4.1 ^bc^	2.14 ± 0.18 ^b^	12.9 ± 0.3 ^b^	29.6 ± 0.5 ^b^	9.71 ± 0.28 ^a^	676 ± 16 ^c^	64.9 ± 2.7 ^ab^
	5	2255 ± 39 ^cd^	72.5 ± 4.1 ^de^	1.76 ± 0.18 ^cd^	12.8 ± 0.3 ^b^	29.0 ± 0.5 ^bc^	8.89 ± 0.28 ^cd^	661 ± 16 ^c^	58.4 ± 2.7 ^cd^
	7	2439 ± 39 ^a^	83.5 ± 4.1 ^b^	2.03 ± 0.18 ^bc^	12.1 ± 0.3 ^c^	28.2 ± 0.5 ^c^	9.57 ± 0.28 ^ab^	693 ± 16 ^b^	66.1 ± 2.7 ^ab^
	10	2459 ± 39 ^a^	73.1 ± 4.1 ^de^	1.71 ± 0.18 ^cd^	10.5 ± 0.3 ^d^	25.3 ± 0.5 ^d^	9.64 ± 0.28 ^ab^	626 ± 16 ^d^	58.9 ± 2.7 ^cd^

Notes: RD, resistant dextrin; RXY, Ruan Xiangyu; Gumminess = hardness × cohesiveness; Chewiness =hardness × cohesiveness × springiness. All data are shown as mean ± SD; number of trials―n = 3, and each rice variety, each RD species, and each RD addition level separately have 30, 30, and 12 data points for UGLM analysis. Different superscript letters represent significant differences (*p* < 0.05) within the column.

**Table 3:** Effect of adding RD on the taste score of cooked rice.

Factors	Levels	Smell (%)	Appearance Structure(%)	Palatability (%)	Taste (%)	Cool Rice Texture(%)	Total Score (%)
Rice	RXY	17.55 ± 0.08 ^a^	17.55 ± 0.09 ^a^	23.08 ± 0.11 ^a^	21.69 ± 0.09 ^a^	4.34 ± 0.06 ^a^	84.20 ± 0.19 ^a^
variety	IP44	16.14 ± 0.08 ^e^	16.06 ± 0.09 ^e^	21.38 ± 0.11 ^e^	18.65 ± 0.09 ^e^	3.79 ± 0.06 ^c^	76.01 ± 0.19 ^h^
RD	BL	16.91 ± 0.08 ^c^	16.85 ± 0.09 ^b^	22.59 ± 0.11 ^b^	20.16 ± 0.09 ^c^	4.10 ± 0.06 ^b^	80.61 ± 0.19 ^d^
species	LGT	16.78 ± 0.08 ^c^	16.76 ± 0.09 ^bc^	21.86 ± 0.11 ^c^	20.18 ± 0.09 ^c^	4.03 ± 0.06 ^b^	79.60 ± 0.19 ^e^
Addition	0	16.38 ± 0.13 ^d^	16.38 ± 0.14 ^d^	21.75 ± 0.17 ^d^	19.50 ± 0.14 ^d^	3.75 ± 0.09 ^c^	77.75 ± 0.31 ^g^
(%)	3	16.63 ± 0.13 ^cd^	16.59 ± 0.14 ^cd^	21.88 ± 0.17 ^cd^	20.09 ± 0.14 ^c^	3.84 ± 0.09 ^c^	79.03 ± 0.31 ^f^
	5	16.84 ± 0.13 ^c^	16.78 ± 0.14 ^bc^	22.19 ± 0.17 ^c^	20.28 ± 0.14 ^c^	4.06 ± 0.09 ^b^	80.16 ± 0.31 ^d^
	7	17.22 ± 0.13 ^b^	16.88 ± 0.14 ^b^	22.56 ± 0.17 ^b^	20.31 ± 0.14 ^c^	4.25 ± 0.09 ^a^	81.22 ± 0.31 ^c^
	10	17.16 ± 0.13 ^b^	17.41 ± 0.14 ^a^	22.75 ± 0.17 ^b^	20.66 ± 0.14 ^b^	4.44 ± 0.09 ^a^	82.38 ± 0.31 ^b^

Notes: RD, resistant dextrin; RXY, Ruan Xiangyu; data are shown as mean ± SD; number of trials―n = 8. The data points for each rice variety, each RD species, and each RD addition level separately are 80, 80, and 32 for UGLM analysis. The different superscript letters represent significant differences (*p* < 0.05) within the column.

**Table 4:** Effects of adding RD on the pasting properties of two varieties of white rice.

Factors	Levels	Peak Viscosity (cP)	Trough Viscosity (cP)	Breakdown Viscosity (cP)	Final Viscosity (cP)	Setback Viscosity (cP)	Peak Time (min)	Pasting Temp. (°C)
Rice	RXY	3541 ± 7 ^f^	2071 ± 11 ^h^	1469 ± 11 ^a^	2751 ± 11 ^h^	680 ± 13 ^h^	5.93 ± 0.02 ^e^	74.36 ± 0.06 ^h^
variety	IP44	3683 ± 7 ^c^	3160 ± 11 ^a^	523 ± 11 ^g^	4993 ± 11 ^a^	1833 ± 13 ^a^	6.37 ± 0.02 ^a^	86.18 ± 0.06 ^a^
RD	BL	3617 ± 7 ^e^	2616 ± 11 ^e^	1001 ± 11 ^d^	3886 ± 11 ^d^	1269 ± 13 ^d^	6.15 ± 0.02 ^c^	80.19 ± 0.06 ^e^
species	LGT	3607 ± 7 ^e^	2615 ± 11 ^e^	992 ± 11 ^d^	3859 ± 11 ^e^	1243 ± 13 ^e^	6.15 ± 0.02 ^c^	80.35 ± 0.06 ^d^
Addition	0	4083 ± 11 ^a^	2803 ± 17 ^b^	1281 ± 17 ^b^	4195 ± 17 ^b^	1393 ± 20 ^b^	5.91 ± 0.02 ^e^	78.80 ± 0.10 ^g^
(%)	3	3872 ± 11 ^b^	2752 ± 17 ^c^	1121 ± 17 ^c^	4069 ± 17 ^c^	1317 ± 20 ^c^	6.09 ± 0.02 ^d^	79.84 ± 0.10 ^f^
	5	3637 ± 11 ^d^	2647 ± 17 ^d^	989 ± 17 ^d^	3913 ± 17 ^d^	1266 ± 20 ^de^	6.18 ± 0.02 ^c^	80.53 ± 0.10 ^c^
	7	3413 ± 11 ^g^	2537 ± 17 ^f^	875 ± 17 ^e^	3738 ± 17 ^f^	1201 ± 20 ^f^	6.24 ± 0.02 ^b^	80.65 ± 0.10 ^c^
	10	3055 ± 11 ^h^	2339 ± 17 ^g^	715 ± 17 ^f^	3446 ± 17 ^g^	1106 ± 20 ^g^	6.33 ± 0.02 ^a^	81.53 ± 0.10 ^b^

Notes: RD, resistant dextrin; RXY, Ruan Xiangyu; all data are shown as mean ± SD; number of trials―n = 3; each rice variety, each RD species, and each RD addition level separately have 30, 30, and 12 data points for UGLM analysis. Different superscript letters represent significant differences (*p* < 0.05) within the column.

**Table 5:** Effect of RD on the thermal characteristics of two varieties of rice on day 0.

Factors	Levels	△H (J/g)	*T*_o_ (°C)	*T*_p_ (°C)	*T*_c_ (°C)	Peak Width (°C)	Peak Height (0.01 mw/mg)
Rice	RXY	6.24 ± 0.07 ^ef^	61.99 ± 0.16 ^f^	70.95 ± 0.06 ^g^	79.33 ± 0.11 ^f^	8.62 ± 0.06 ^a^	9.79 ± 0.15 ^g^
variety	IP44	7.04 ± 0.07 ^c^	72.86 ± 0.16 ^a^	77.62 ± 0.06 ^a^	83.85 ± 0.11 ^a^	5.26 ± 0.06 ^f^	17.09 ± 0.15 ^a^
RD	BL	6.38 ± 0.07 ^e^	67.61 ± 0.16 ^bc^	74.26 ± 0.06 ^cd^	81.54 ± 0.11 ^d^	6.91 ± 0.06 ^c^	13.17 ± 0.15 ^e^
species	LGT	6.91 ± 0.07 ^c^	67.24 ± 0.16 ^d^	74.31 ± 0.06 ^c^	81.63 ± 0.11 ^d^	6.96 ± 0.06 ^c^	13.72 ± 0.15 ^cd^
Addition	0	8.05 ± 0.11 ^a^	66.83 ± 0.25 ^e^	74.12 ± 0.10 ^de^	82.17 ± 0.18 ^b^	7.33 ± 0.09 ^b^	15.08 ± 0.24 ^b^
(%)	3	7.36 ± 0.11 ^b^	67.49 ± 0.25 ^bcd^	74.07 ± 0.10 ^e^	81.99 ± 0.18 ^bc^	7.22 ± 0.09 ^b^	14.01 ± 0.24 ^c^
	5	6.59 ± 0.11 ^d^	67.27 ± 0.25 ^cde^	74.19 ± 0.10 ^cde^	81.73 ± 0.18 ^cd^	6.85 ± 0.09 ^cd^	13.37 ± 0.24 ^de^
	7	6.08 ± 0.11 ^f^	67.54 ± 0.25 ^bcd^	73.42 ± 0.10 ^f^	81.19 ± 0.18 ^e^	6.73 ± 0.09 ^de^	13.19 ± 0.24 ^e^
	10	5.14 ± 0.11 ^g^	67.99 ± 0.25 ^b^	74.63 ± 0.10 ^b^	80.85 ± 0.18 ^e^	6.55 ± 0.09 ^e^	11.58 ± 0.24 ^f^

Notes: RD, resistant dextrin; RXY, Ruan Xiangyu; △H, T_o_, T_p_, and T_c_ are the enthalpy, onset temperature, peak temperature, and conclusion temperature of gelatinisation, respectively. All data are shown as mean ± SD; number of trials―n = 3;each rice variety, each RD species, and each RD addition level separately have 30, 30, and 12 data points for UGLM analysis. Different superscript letters represent significant differences (*p* < 0.05) within the column.

**Table 6:** Effect of RD on the thermal properties of two varieties of rice on day 21.

Factors	Levels	△H (J/g)	*T*_o_ (°C)	*T*_p_ (°C)	*T*_c_ (°C)	Peak Width (°C)	Peak Height (0.01 mw/mg)	Amylopectin Ageing (%)
Rice	RXY	0.60 ± 0.05 ^g^	54.09 ± 0.94 ^a^	59.69 ± 0.32 ^a^	65.03 ± 0.29 ^d^	8.09 ± 0.14 ^f^	1.51 ± 0.19 ^c^	9.59 ± 1.45 ^e^
variety	IP44	4.41 ± 0.05 ^a^	47.49 ± 0.94 ^d^	58.95 ± 0.32 ^b^	66.49 ± 0.29 ^ab^	10.39 ± 0.14 ^a^	6.20 ± 0.19 ^a^	64.08 ± 1.45 ^a^
RD	BL	2.44 ± 0.05 ^e^	50.17 ± 0.94 ^bc^	59.35 ± 0.32 ^ab^	65.59 ± 0.29 ^cd^	9.47 ± 0.14 ^bc^	3.94 ± 0.19 ^b^	38.00 ± 1.45 ^c^
species	LGT	2.56 ± 0.05 ^d^	51.42 ± 0.94 ^bc^	59.28 ± 0.32 ^ab^	65.94 ± 0.29 ^bc^	9.01 ± 0.14 ^de^	3.78 ± 0.19 ^b^	35.67 ± 1.45 ^cd^
Addition	0	2.91 ± 0.08 ^b^	52.55 ± 1.49 ^ab^	58.07 ± 0.50 ^c^	67.05 ± 0.46 ^a^	9.87 ± 0.22 ^b^	4.01 ± 0.31 ^b^	34.49 ± 2.29 ^cd^
(%)	3	2.73 ± 0.08 ^c^	51.90 ± 1.49 ^abc^	59.90 ± 0.50 ^a^	65.63 ± 0.46 ^cd^	9.23 ± 0.22 ^c^	3.94 ± 0.31 ^b^	35.61 ± 2.29 ^cd^
	5	2.49 ± 0.08 ^de^	49.47 ± 1.49 ^cd^	59.40 ± 0.50 ^ab^	65.48 ± 0.46 ^cd^	9.38 ± 0.22 ^cd^	3.61 ± 0.31 ^b^	35.77 ± 2.29 ^cd^
	7	2.21 ± 0.08 ^f^	51.08 ± 1.49 ^bc^	59.98 ± 0.50 ^a^	65.45 ± 0.46 ^cd^	8.86 ± 0.22 ^e^	3.92 ± 0.31 ^b^	34.14 ± 2.29 ^d^
	10	2.17 ± 0.08 ^f^	48.98 ± 1.49 ^cd^	59.25 ± 0.50 ^ab^	65.22 ± 0.46 ^cd^	8.88 ± 0.22 ^e^	3.82 ± 0.31 ^b^	44.15 ± 2.29 ^b^

Notes: RD, resistant dextrin; RXY, Ruan Xiangyu; △H, T_o_, T_p_, and T_c_ are the enthalpy, onset temperature, peak temperature, and conclusion temperature of gelatinisation, respectively. All data are shown as mean ± SD; number of trials―n = 3;each rice variety, each RD species, and each RD addition level separately have 30, 30, and 12 data points for UGLM analysis. Different superscript letters represent significant differences (*p* < 0.05) within the column.

**Table 7:** Effect of RD on the thermo-mechanical characteristics of milled rice flour dough.

Factors	Levels	DDT (min)	DST (min)	C1 (Nm)	C2 (0.1 Nm)	C3 (Nm)
Rice	RXY	1.146 ± 0.067 ^a^	1.350 ± 0.331 ^b^	1.795 ± 0.048 ^b^	5.37 ± 0.13 ^a^	1.165 ± 0.015 ^g^
variety	IP44	0.935 ± 0.067 ^b^	2.115 ± 0.331 ^a^	0.943 ± 0.048 ^f^	3.43 ± 0.13 ^e^	1.823 ± 0.015 ^a^
RD	BL	1.027 ± 0.067 ^ab^	1.604 ± 0.331 ^ab^	1.343 ± 0.048 ^de^	4.33 ± 0.13 ^c^	1.467 ± 0.015 ^ef^
species	LGT	1.053 ± 0.067 ^ab^	1.861 ± 0.331 ^ab^	1.395 ± 0.048 ^cd^	4.48 ± 0.13 ^bc^	1.522 ± 0.015 ^c^
Addition	0	1.078 ± 0.107 ^ab^	1.301 ± 0.522 ^ab^	1.957 ± 0.076 ^a^	5.36 ± 0.21 ^a^	1.566 ± 0.023 ^b^
(%)	3	1.034 ± 0.107 ^b^	1.548 ± 0.522 ^ab^	1.480 ± 0.076 ^c^	4.68 ± 0.21 ^b^	1.513 ± 0.023 ^cd^
	5	0.947 ± 0.107 ^b^	1.501 ± 0.522 ^ab^	1.221 ± 0.076 ^e^	4.69 ± 0.21 ^b^	1.472 ± 0.023 ^def^
	7	0.980 ± 0.107 ^ab^	1.345 ± 0.522 ^ab^	1.259 ± 0.076 ^e^	3.84 ± 0.21 ^d^	1.485 ± 0.023 ^cde^
	10	1.163 ± 0.107 ^a^	2.969 ± 0.522 ^ab^	0.928 ± 0.076 ^f^	3.44 ± 0.21 ^de^	1.435 ± 0.023 ^f^
Factors	Levels	C4 (Nm)	C5 (Nm)	C1–Cs (0.1 Nm)	C3–C4 (0.01 Nm)	C3/C4
Rice	RXY	1.003 ± 0.016 ^e^	1.693 ± 0.023 ^c^	7.32 ± 0.54 ^b^	16.26 ± 0.42 ^a^	1.165 ± 0.005 ^b^
variety	IP44	1.781 ± 0.016 ^a^	2.703 ± 0.023 ^a^	2.25 ± 0.54 ^e^	4.21 ± 0.42 ^g^	1.024 ± 0.005 ^f^
RD	BL	1.363 ± 0.016 ^d^	2.166 ± 0.023 ^b^	4.81 ± 0.54 ^c^	10.35 ± 0.42 ^c^	1.095 ± 0.005 ^c^
species	LGT	1.421 ± 0.016 ^b^	2.230 ± 0.023 ^b^	4.75 ± 0.54 ^c^	10.11 ± 0.42 ^c^	1.093 ± 0.005 ^c^
Addition	0	1.394 ± 0.025 ^bcd^	2.212 ± 0.036 ^b^	8.90 ± 0.86 ^a^	17.12 ± 0.66 ^a^	1.155 ± 0.008 ^a^
(%)	3	1.388 ± 0.025 ^bcd^	2.196 ± 0.036 ^b^	5.05 ± 0.86 ^c^	12.49 ± 0.66 ^b^	1.116 ± 0.008 ^b^
	5	1.386 ± 0.025 ^bcd^	2.213 ± 0.036 ^b^	3.30 ± 0.86 ^d^	8.64 ± 0.66 ^d^	1.080 ± 0.008 ^d^
	7	1.413 ± 0.025 ^bc^	2.211 ± 0.036 ^b^	4.55 ± 0.86 ^cd^	7.20 ± 0.66 ^e^	1.066 ± 0.008 ^de^
	10	1.378 ± 0.025 ^cd^	2.159 ± 0.036 ^b^	2.11 ± 0.86 ^e^	5.72 ± 0.66 ^f^	1.053 ± 0.008 ^e^
Factors	Levels	C5–C4 (0.1 Nm)	Alpha (−0.01 Nm)	Beta (0.01 Nm)	Gama (−0.01 Nm)	
Rice	RXY	6.901 ± 0.120 ^d^	8.21 ± 0.19 ^a^	15.51 ± 0.91 ^e^	2.86 ± 0.27 ^bc^	
variety	IP44	9.223 ± 0.120 ^a^	5.18 ± 0.19 ^f^	30.65 ± 0.91 ^a^	2.75 ± 0.27 ^bc^	
RD	BL	8.032 ± 0.120 ^bc^	6.54 ± 0.19 ^de^	23.47 ± 0.91 ^bc^	3.24 ± 0.27 ^a^	
species	LGT	8.091 ± 0.120 ^bc^	6.85 ± 0.19 ^cd^	22.69 ± 0.91 ^cd^	2.37 ± 0.27 ^c^	
Addition	0	8.173 ± 0.190 ^bc^	8.37 ± 0.31 ^b^	21.67 ± 1.43 ^cd^	3.17 ± 0.42 ^ab^	
(%)	3	8.084 ± 0.190 ^bc^	7.25 ± 0.31 ^c^	23.62 ± 1.43 ^bcd^	3.60 ± 0.42 ^a^	
	5	8.271 ± 0.190 ^b^	6.47 ± 0.31 ^de^	24.00 ± 1.43 ^bc^	2.62 ± 0.42 ^bc^	
	7	7.974 ± 0.190 ^bc^	6.12 ± 0.31 ^e^	25.27 ± 1.43 ^b^	2.33 ± 0.42 ^c^	
	10	7.807 ± 0.190 ^c^	5.27 ± 0.31 ^f^	20.85 ± 1.43 ^d^	2.32 ± 0.42 ^c^	

Note: RD, resistant dextrin; RXY, Ruan Xiangyu; DDT, dough development time; DST, dough stability time; C1, maximum consistency; C2, minimum consistency; C3, maximum gelatinisation torque; C4, persistent viscosity (gel thermal stability); C5, setback end torque; C1–Cs, protein weakness degree; C3–C4, starch breakdown torque; C3/C4, endogenous amylase activity; C5–C4, starch setback torque; Alpha (α), heating speed; Beta (β), gelatinisation rate; Gama (γ), enzymatic degradation rate. All data are shown as mean ± SD; number of trials―n = 3; the data points of each rice variety, each RD species, and each RD addition level separately are 30, 30, and 12 for UGLM analysis. Different superscript letters represent significant differences (*p* < 0.05) within the column.

**Table 8:** Modification effect of RD on the starch crystallinity and protein conformation of cooked white rice.

Factors	Levels	R_1022/995_	R_1047/1022_	R_1068/1022_	
Rice	RSY	1.089 ± 0.003 ^ab^	0.908 ± 0.003 ^ab^	0.774 ± 0.006 ^a^	
variety	IP44	1.083 ± 0.003 ^b^	0.905 ± 0.003 ^ab^	0.755 ± 0.006 ^b^	
RD	BL	1.083 ± 0.003 ^b^	0.907 ± 0.003 ^ab^	0.766 ± 0.006 ^abc^	
species	LGT	1.089 ± 0.003 ^ab^	0.906 ± 0.003 ^ab^	0.763 ± 0.006 ^abc^	
Addition	0	1.074 ± 0.005 ^c^	0.901 ± 0.004 ^b^	0.761 ± 0.010 ^abc^	
(%)	3	1.088 ± 0.005 ^ab^	0.904 ± 0.004 ^ab^	0.756 ± 0.010 ^bc^	
	5	1.084 ± 0.005 ^bc^	0.911 ± 0.004 ^a^	0.773 ± 0.010 ^ab^	
	7	1.091 ± 0.005 ^ab^	0.912 ± 0.004 ^a^	0.774 ± 0.010 ^ab^	
	10	1.096 ± 0.005 ^a^	0.904 ± 0.004 ^ab^	0.761 ± 0.010 ^abc^	
Factors	Levels	Beta-sheet (%)	Random coils (%)	Alpha-helix (%)	Beta-turn (%)
Rice	RSY	53.217 ± 0.245 ^a^	15.445 ± 0.062 ^e^	15.543 ± 0.072 ^e^	15.795 ± 0.124 ^d^
variety	IP44	51.541 ± 0.245 ^d^	15.924 ± 0.062 ^a^	16.359 ± 0.072 ^a^	16.177 ± 0.124 ^ab^
RD	BL	52.136 ± 0.245 ^c^	15.735 ± 0.062 ^bc^	16.005 ± 0.072 ^bc^	16.124 ± 0.124 ^bc^
species	LGT	52.621 ± 0.245 ^bc^	15.634 ± 0.062 ^cd^	15.897 ± 0.072 ^cd^	15.848 ± 0.124 ^d^
Addition	0	52.412 ± 0.383 ^bc^	15.516 ± 0.098 ^de^	15.890 ± 0.113 ^cd^	16.183 ± 0.197 ^abc^
(%)	3	52.393 ± 0.383 ^bc^	15.801 ± 0.098 ^ab^	16.018 ± 0.113 ^bc^	15.788 ± 0.197 ^d^
	5	52.552 ± 0.383 ^bc^	15.682 ± 0.098 ^cd^	15.946 ± 0.113 ^bcd^	15.820 ± 0.197 ^cd^
	7	53.051 ± 0.383 ^ab^	15.548 ± 0.098 ^de^	15.753 ± 0.113 ^d^	15.649 ± 0.197 ^d^
	10	51.485 ± 0.383 ^d^	15.877 ± 0.098 ^ab^	16.148 ± 0.113 ^b^	16.491 ± 0.197 ^a^

Notes: RD, resistant dextrin; RXY, Ruan Xiangyu; R_1022/995_ and R_1047/1022_ display the extent of short sequences at the surface of a starch granule, and R_1068_/_1022_ gives the possible interaction between a starch granule and a protein. All data are shown as mean ± SD; number of trials―n = 3; the data points of each rice cultivar, each RD species, and each RD addition level separately are 30, 30, and 12 for UGLM analysis. The different superscript letters represent significant differences (*p* < 0.05) in the same column.

**Table 9:** The properties of the two rice samples.

Rice Variety	Moisture Content (%)	Kernel Length-to-Width Ratio	Free Fatty Acid (mgKOH/100g)	Amylose (%)	Protein (%)
RXY	13.37 ± 0.06 ^b^	1.61 ± 0.01 ^a^	15.52 ± 0.75 ^a^	9.00 ± 0.53 ^b^	11.33 ± 0.15 ^a^
IP44	12.82 ± 0.06 ^a^	1.97 ± 0.06 ^a^	16.21 ± 0.74 ^b^	23.10 ± 0.10 ^a^	11.23 ± 0.12 ^b^

Note: RXY is cv. Ruan Xiangyu; the data are shown as mean ± SD; number of trials―n = 3. Different superscript letters mean significant difference (*p* < 0.05) in the same column.

## Data Availability

The original contributions presented in this study are included in this article; other inquiries can be directed to the corresponding author.

## Supplementary Materials

The following supporting information can be downloaded at: https://www.mdpi.com/article/10.3390/gels12060516/s1, Figure S1. The pasting curves of RD-added rice flour; Figure S2. The 0-day thermal property curves of RD-added rice flour; Figure S3. The 21-day thermal property curves of RD-added rice paste; Figure S4. The FTIR spectra curves of cooked rice with RD added; Figure S5. The range scaling normalised FTIR spectra curves of cooked rice with RD addition.
