# Supplementary material for: Effect of Resistant Dextrin on the Functional, Thermal and Structural Properties of Cooked Chinese Rice
Source: Gels. 2026 Jun 10;12(6):516. doi: 10.3390/gels12060516 (PMC13298026; doi:10.3390/gels12060516)
Supplement: Supplementary file 1 [file gels-12-00516-s001.zip › gels-4247531-supplementary.pdf]

Attachment file

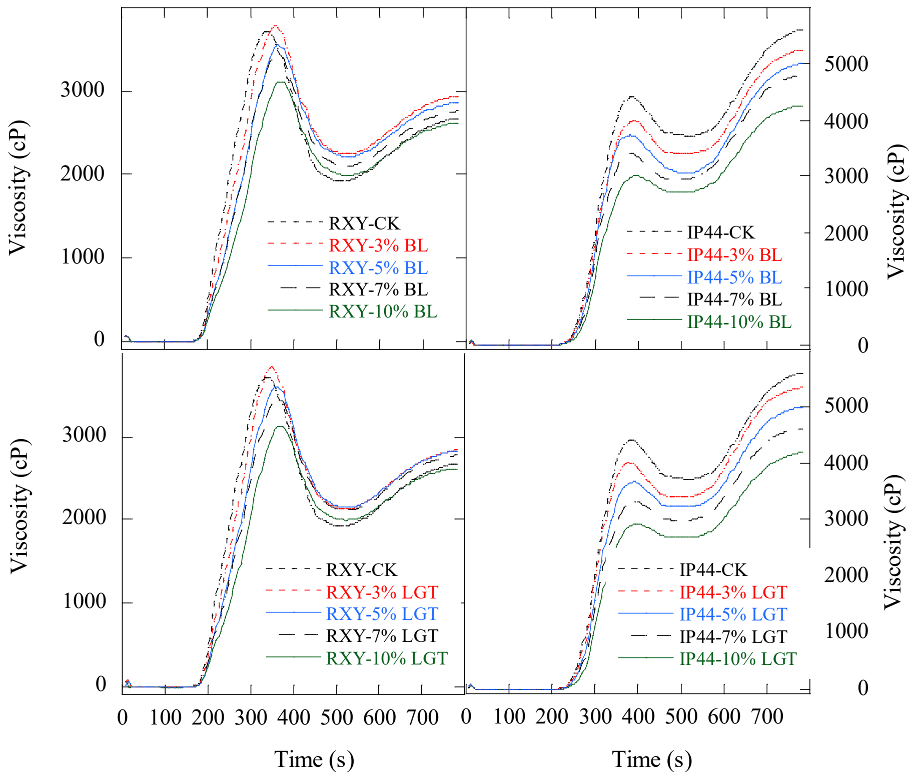

Figure S1. The pasting curves of RD-added rice flour.

Note: RXY and IP44 are two rice varieties; BL and LGT are two species of resistant dextrin; CK is the control sample without RD addition.

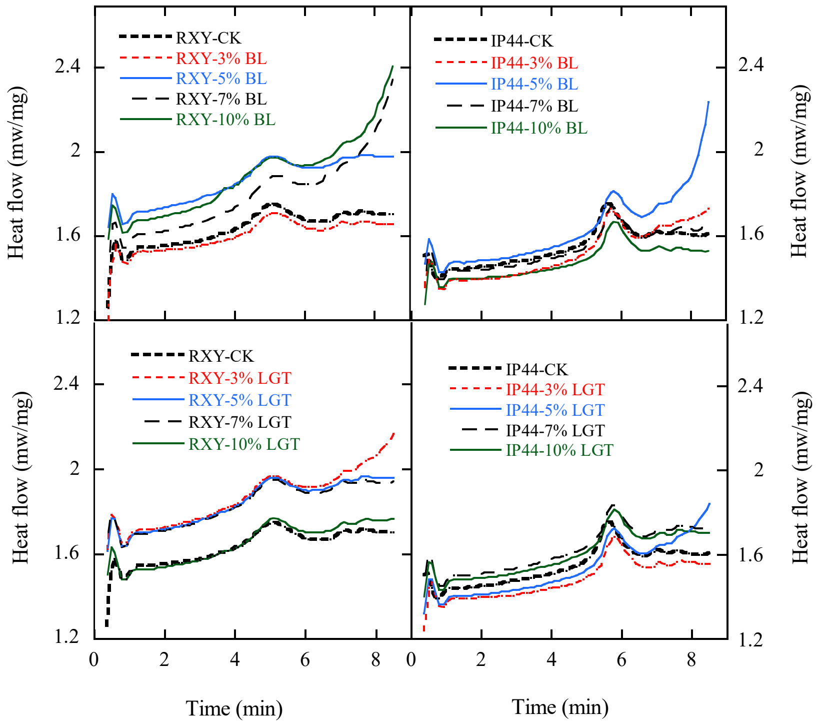

Figure S2. The 0-day thermal property curves of RD-added rice flour.

Note: RXY and IP44 are two rice varieties; BL and LGT are two species of resistant dextrin; CK is the control sample without RD addition.

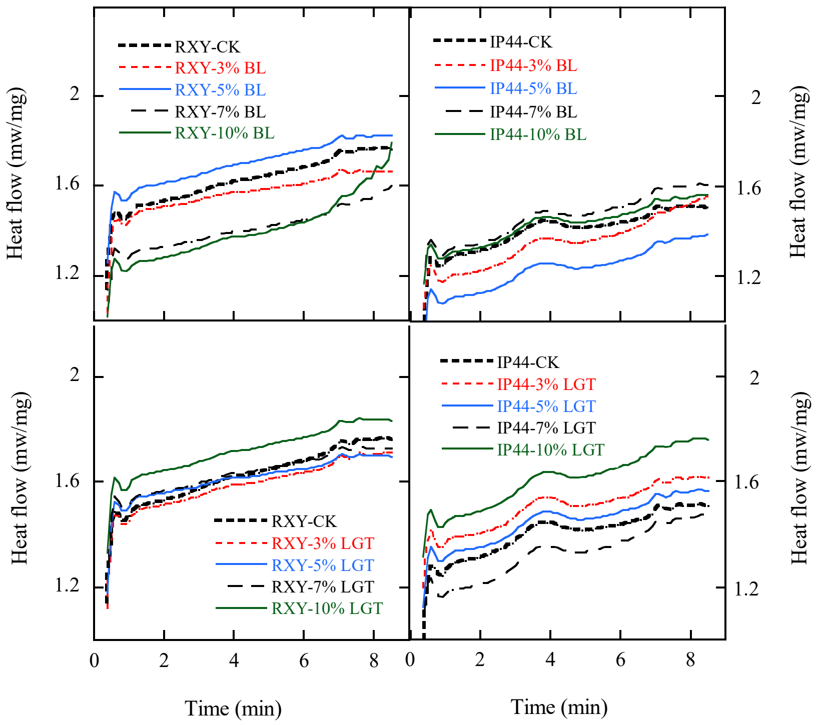

Figure S3. The 21-day thermal property curves of RD-added rice paste.

Note: RXY and IP44 are two rice varieties; BL and LGT are two species of resistant dextrin; CK is the control sample without RD addition.

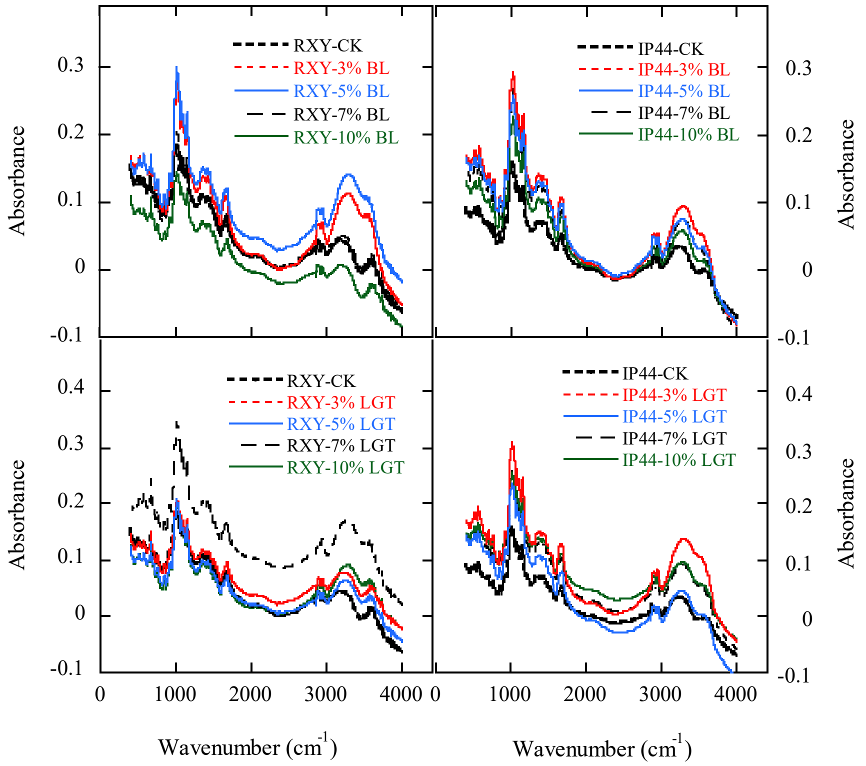

Figure S4. The FTIR spectra curves of cooked rice with RD addition.

Note: RXY and IP44 are two rice varieties; BL and LGT are two species of resistant dextrin; CK is the control sample without RD addition.

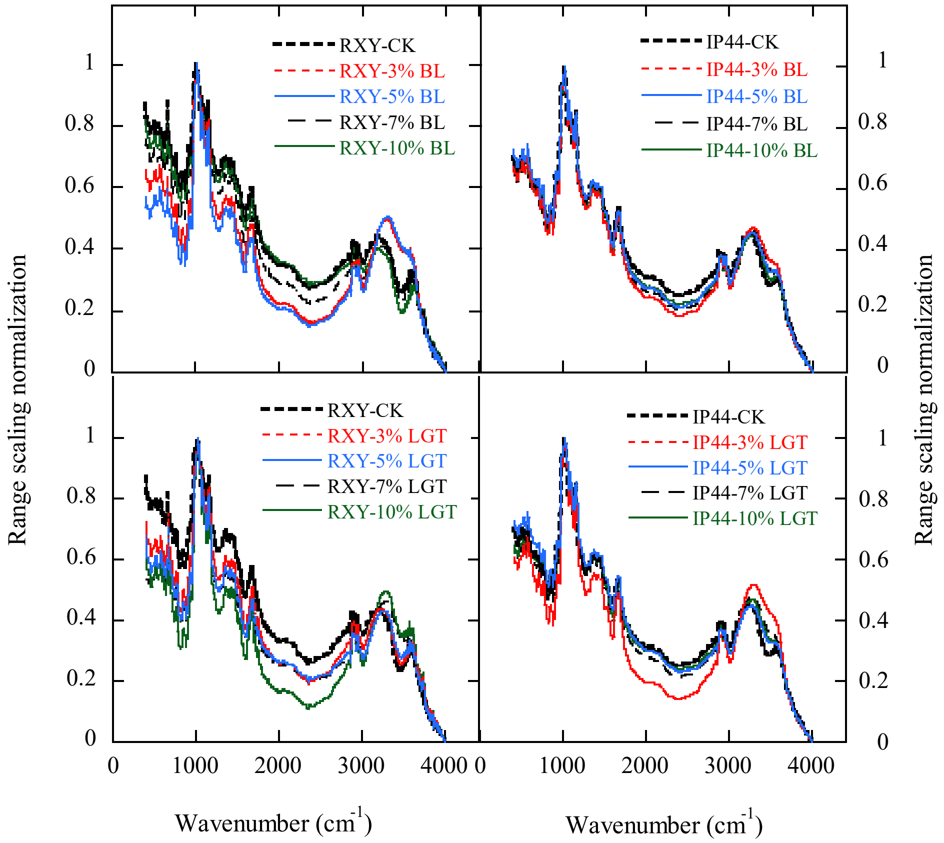

Figure S5. The range scaling normalised FTIR spectra curves of cooked rice with RD addition. Note: RXY and IP44 are two rice varieties; BL and LGT are two species of resistant dextrin; CK is the control sample without RD addition.
